# Lipid metabolism and tumor immunotherapy

**DOI:** 10.3389/fcell.2023.1187989

**Published:** 2023-05-16

**Authors:** Yue Wang, Zongjin Guo, Adamu Danbala Isah, Shuangwei Chen, Yongfei Ren, Huazhong Cai

**Affiliations:** ^1^ School of Medicine, Jiangsu University, Zhenjiang, China; ^2^ Cancer Institute of Jiangsu University, Affiliated Hospital of Jiangsu University, Zhenjiang, China; ^3^ Department of Emergency, Affiliated Hospital of Jiangsu University, Zhenjiang, China; ^4^ Department of Interventional Radiology, The University of Hong Kong-Shenzhen Hospital, Shenzhen, China

**Keywords:** lipid metabolism, tumor microenvironment, immunotherapy, combination therapy, immune cells

## Abstract

In recent years, the relationship between lipid metabolism and tumour immunotherapy has been thoroughly investigated. An increasing number of studies have shown that abnormal gene expression and ectopic levels of metabolites related to fatty acid synthesis or fatty acid oxidation affect tumour metastasis, recurrence, and drug resistance. Tumour immunotherapy that aims to promote an antitumour immune response has greatly improved the outcomes for tumour patients. However, lipid metabolism reprogramming in tumour cells or tumour microenvironment-infiltrating immune cells can influence the antitumour response of immune cells and induce tumor cell immune evasion. The recent increase in the prevalence of obesity-related cancers has drawn attention to the fact that obesity increases fatty acid oxidation in cancer cells and suppresses the activation of immune cells, thereby weakening antitumour immunity. This article reviews the changes in lipid metabolism in cells in the tumour microenvironment and describes the relationship between lipid metabolism reprogramming in multiple cell types and tumour immunotherapy.

## Introduction

In recent years, tumour immunotherapy has been rapidly developed and remarkable clinical efficacy has been achieved (Li et al., 2019; Wei et al., 2021). The application of immune checkpoint inhibitors, such as PD-1 and PDL-1, has shown efficacy against malignant melanoma, non-small cell lung cancer (NSCLC), and other tumours (Coleman et al., 2020). The growth environment of tumours is different from that of normal tissue. Various cell types of cells populate the TME, and they engage in extensive interactions with each other. Immune cells, including T cells, dendritic cells (DCs), tumour-associated macrophages (TAMs), polymorphonuclear neutrophils (PMNs), and myeloid-derived suppressor cells (MDSCs) (Wei et al., 2021), play essential roles in the malignant progression of tumours. Various changes in the tumour immune microenvironment facilitate tumour cell escape tumour immune surveillance (Gonzalez et al., 2018; Duan et al., 2019; Peng et al., 2019).

The growth of tumours requires a variety of nutrients, and to obtain additional nutrients, three primary metabolic pathways, namely, sugar metabolism, amino acid metabolism, and lipid metabolism pathways in cells within the TME are changed to promote tumour growth and spread. Lipids are required for the rapid growth and proliferation of tumour cells. Uncontrolled *de novo* fatty acid synthesis is an important metabolic characteristic of cancer cells (Li et al., 2019; Mo et al., 2019; Wei et al., 2021). Tumour cells need lipids to form cell membranes and undergo cell division, and intermediates in lipid metabolism pathways are also involved in regulating tumour cell proliferation and growth signalling pathways. Moreover, changes in lipid metabolism in immune cells is closely related to the cell effects on the tumour immune microenvironment (Corn et al., 2020). Therefore, the association between lipid metabolism in immune cells in the TME promote the proliferation of tumour cells. Targeting the lipid metabolism pathways in TME immune cells in combination with tumour immunotherapy is a strategy to attenuate tumour growth.

## Lipid metabolism and tumour growth

Lipid metabolism plays a central role in many biological processes. Lipid-related enzymes regulate the synthesis of fatty acids (FAs) in a process mediated by upstream signalling networks triggered by cell nutritional status. In the TME, lipid metabolism is driven by oncogenes, tumour suppressors, and other factors that enable tumour cells to adapt to the hypoxia and nutrient deprivation in the TME (8-10). The key transcription factors regulating lipid metabolism include sterol-regulatory element-binding proteins (SREBPs), liver X receptor (LXR), and peroxisome proliferator-activated receptors (PPARs). Lipid metabolism in the TME mainly provides phospholipids sufficient for formation of the plasma membrane, and therefore, abnormal lipid metabolism increases conducive to tumour cell permeability. In the hypoxic TME, the rates of the conversion of pyruvate to lactic acid and the subsequent production of acetyl coenzyme A (AcCoA) are reduced, and under these conditions, tumour cells can only obtain enough AcCoA through glutamine catabolism. AcCoA is the main intermediate product of various metabolic processes. It plays a vital role in the biosynthesis of FAs, cholesterol, and fatty acid oxidation (FAO) products. Carnitine palmitoyl transferase 1 (CPT1), the rate-limiting enzyme in FAO, shows therapeutic potential as a target of anticancer drugs (Manzo et al., 2020). Although not required for FAO, fatty acid synthase (FASN) is an essential enzyme in fatty acid synthesis and it has also become a hotspots in anticancer drug research (Zhu and Thompson, 2019).

Adipose tissue is a component of the cell membrane store energy and undergo oxidation. Adipose tissue is categorized into two subtypes, white adipose tissue (WAT) and brown adipose tissue. WAT is the main form of fat storage in the body, while brown adipose tissue primarily plays a role in thermogenesis (Moukarzel et al., 2022). Studies have shown that when WAT turns brown, cancer patients develop cachexia, and fat cells are reshaped. Adipocytes produce adipocytokines and adipokines, which are essential for tumour angiogenesis. They stimulate tumour cells and mediate their proliferation and migration to promote tumour progression (Rathmell, 2021; Moukarzel et al., 2022).

**FIGURE 1:**
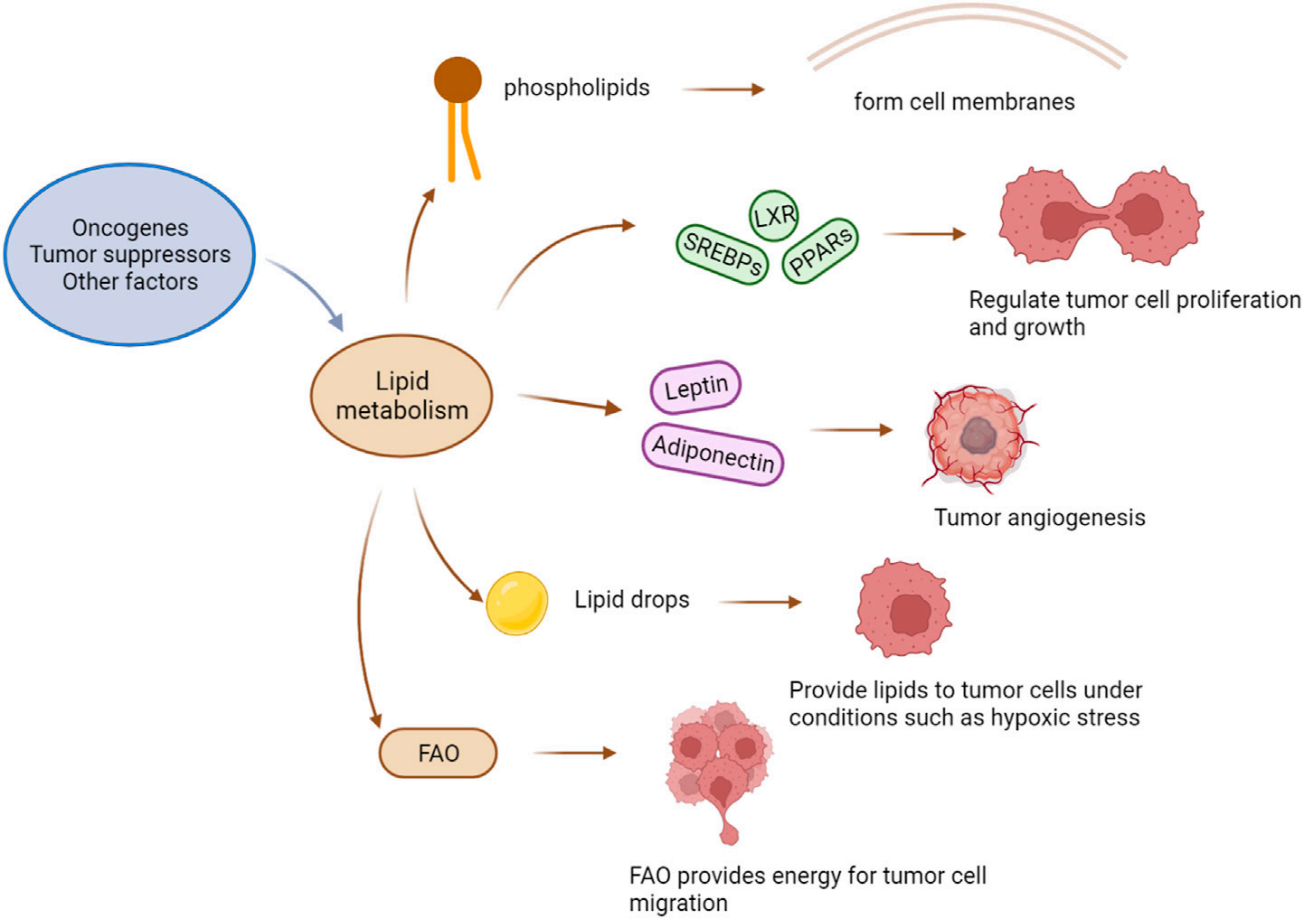
The effect of lipid metabolism on a tumor. Factors such as oncogenes and tumor suppressors drive changes in lipid metabolism, and the phospholipids produced by lipid metabolism constitute the cell membrane of tumor cells. Key transcription factors that regulate metabolisms, such as SREBPs (sterol-regulatory element binding proteins), LXRs (liver X-activated receptors), and PPARs (peroxisome proliferators-activated receptors), regulate the proliferation and growth of tumor cells. Adipocytokines (Leptin and Adiponectin) promote tumor angiogenesis. Lipid droplets can provide lipids for tumor cells under extreme conditions. FAO provides energy for tumor cell migration.

Lipids can be obtained through biosynthesis and from diet. Compared with normal cells that rely on dietary lipid intake, tumour cells rely on exogenous lipids and endogenous lipids, including those stored in lipid droplets (LDs). LDs are the primary lipid storage organelles in eukaryotic cells (Bosch et al., 2020), storing a large number of triglycerides and cholesterol esters and providing lipids for tumour cells under stress conditions such as hypoxia (Petan, 2020). In addition to storing lipids, LDs are additional lipid sources for sustaining FAO under nutrient-deprivation conditions. For example, the TME cannot always provide sufficient nutrients, and when nutrients are in adequate supply, tumour cells continue to synthesize new FAs and store a large number of FAs in the forms of triglycerides and LDs; then, when the nutrient supply is insufficient, tumour cells quickly release free FAs from LDs for structural lipid synthesis and undergo lipid catabolism and energy generation via accelerated FAO, promoting their rapid growth even under harsh conditions. Hence, many researchers are committed to studying drugs targeting FAO, such as rate-limiting enzyme CPT1 inhibitors (Figure 1).

## Lipid metabolism and tumor immune microenvironment

### T cells

Naive T cells develop and mature in the thymus before entering the circulatory system. After antigen stimulation, naive T cells differentiate into effector T cells and long-lived memory T cells (Chapman et al., 2020). Effector T cells include regulatory T cells, helper T cells, and cytotoxic T cells. Effector T cells shows specific affinity for certain antigens, including antigens invading host cells. Regulatory T cells mediate and promote immunosuppression and tolerogenic responses through contact-dependent and contact-independent mechanisms. Helper T cells promote protective immunity against intracellular pathogens; however, they can also potentiate allergic responses and asthma and promote autoimmune and inflammatory diseases. Cytotoxic T cells can directly kill infected and transformed cells by secreting perforin and granzyme. Central memory T cells rapidly proliferate and differentiate into effector T cells following antigen stimulation. Effector memory T cells provide immediate protection against initial antigen invasion by rapidly producing effector cytokines and engaging in signalling pathways. At a tumour site, the antitumour immune response mediated by T cells is regulated by molecular interactions with immune checkpoint proteins, resulting in the possible inhibition of tumour cell elimination. T cells in the TME are also called tumour-infiltrating lymphocytes (TILs). Compared to that in primitive T cells and effector T cells, the dependence of memory T cells on glycolysis is reduced, and they increasingly depend on mitochondrial FAO. Memory T cell demand for FAO differs, with the that in specific tissues significantly increased. Lipid metabolism regulates T-cell-mediated immune responses by inducing T-cell differentiation, activation, and migration. In recent years, many experiments have linked lipid metabolism with immune cell function (Table 1), and therefore, efforts to mediate their functions in combination has led to better anticancer effects (Sag et al., 2015; Yang et al., 2016; Liu et al., 2019; Paget et al., 2019; Veglia et al., 2019; Li et al., 2020; Ugolini et al., 2020; Liu et al., 2021a; Lim et al., 2021).

**TABLE 1:** Targeted inhibition of lipid metabolism in cancer immunotherapy.

Target object	The main function	Cancer type	Associated immune cells	References
Acyl coenzyme A-cholesterol acyltransferase (ACAT)	Catalyze synthesis of cholesterol esters from free cholesterol and long-chain fatty acids	Melanoma	CD8^+^ T cells	Yang et al. (2016), Li et al. (2020)
IVA phospholipase A_2_	Elevated cPLA_2_α expression is required for LD accumulation and senescence induction in T cells	Melanoma and breast cancer	Tregs	Liu et al. (2021a)
ATP-binding Cassette Transporter G1 (ABCG1)	Promote cholesterol efflux from cells and regulates intracellular cholesterol homeostasis	Bladder carcinoma and melanoma	Macrophages	Sag et al. (2015)
Monosialoganglioside GM3 and disialogangliosi-de GD3	Activate iNKT cells in a CD1d-dependent manner	-	iNKT cells and DC	Paget et al. (2019)
SREBP	Coordinates cellular programs for lipid synthesis and inhibitory receptor signaling in Tregs	Colon adenocarcinoma and melanoma	Tregs	Liu et al. (2019), Lim et al. (2021)
Myeloperoxidase (MPO)	Have a strong effect on cross-presentation by DCs	Lewis lung cancer	PMN-MDSC	Ugolini et al. (2020)
Fatty acid transport protein 2 (FATP2)	Regulate the immunosuppressive function of PMN-MDSCs	Lymphoma, colon carcinoma, and pancreatic cancer	PMN-MDSC	Veglia et al. (2019)

In recent years, some studies have found that by targeting the intermediate products of lipid metabolism, inhibiting lipid metabolism indirectly affects immune cells, which is beneficial to the treatment of cancer.

CD8^+^ T cells differentiate into cytotoxic T cells (CTLs) after activation. In the process of tumour invasion, naive CD8^+^ T cells differentiate into CD8^+^ effector T cells (TEFFs) and can further differentiate into cytotoxic CD8^+^ T cells and memory CD8^+^ T cells to play a targeted role in tumour sites (Li and Li, 2020). The activation of CTLs is the key to antitumour immunity. After CTL activation, the metabolism in CTLs changes; specifically, three major metabolic pathways are upregulated to promote rapid cell proliferation. Because tumour-specific antigen recognition enables TEFFs to induce cell death through perforin, granzyme, and Fas-Fas ligand signaling to destroy cancer cells, their activation improves patient treatment prognosis. Endogenous and exogenous signaling regulates the metabolism of CD8^+^ T cells, the metabolic state of which determines the antitumour immune response and biosynthesis of CD8^+^ T cells. In a mouse melanoma model, cholesterol esterification was achieved through the action of the cholesterol acyltransferase (ACAT) inhibitor avasimibe, which enhanced the effector function and proliferation of CD8^+^ T cells. In addition, ACAT1-deficient CD8^+^ T cells showed better control over the growth and metastasis of melanoma tumours. Some studies have shown that combination therapy with an ACAT inhibitor and an anti-PD-1 antibody shows promise in improving antitumour effects than have been achieved with monotherapy (Li et al., 2020). Some studies have reported on the metabolism of CD8^+^ T cells *in vivo* on the basis of isotope labelling. Mice bearing tumours for 3 days were inoculated with AdC68-gDMelapoly and AdC68-gDE7, and then, they were given ^13^C6-Glc or ^13^C16-palmitate after 14 or 30 days. Compared to the mice bearing tumours for 14 days, the mice bearing tumours for 30th days showed decreased intensity of signals emitted from glycolytic intermediates in CD8^+^ T cells, while the types of markers and the ketone and acylcarnitine levels in CD8^+^ T cells increased. Ultimately, TILs exhibited enhanced FA catabolism (Zhang et al., 2017).

Ma, X et al. proposed that reducing cholesterol can enhance T-cell-based immunotherapy outcomes and restore T-cell function. They stained tumour-infiltrating T cells in a murine melanoma model, sorted the cells, and analysed the apoptosis, cytotoxicity, and proliferation rates. The results showed that the expression level of immune checkpoints (PD-1 and 2B4) on CD8^+^ T cells was positively correlated with the total cholesterol content. Further experiments revealed that tumour-specific CD8^+^ T cells, after homing to a tumour, absorbed accumulate cholesterol, which accumulated, leading to T-cell exhaustion, which increased the expression of immune checkpoint genes. A series of follow-up analyses were performed, including cleaved caspase 3 staining and microarray analysis of CD8^+^ T cells. The results confirmed that cholesterol induced T-cell apoptosis by driving the activation of the ER stress sensor XBP1. In addition, a XBP1 inhibitor significantly enhanced the antitumour activity of CD8^+^ T cells (Ma et al., 2019).

In vaccine-induced advanced tumour CD8^+^ TILs, increased uptake of FAs and the FAO rate-limiting enzyme CPT1a promoted metabolism conversion. The increasing abundance of free FAs in B16Braf_V600E_ tumour interstitial fluid further supported the suggestion that FA catabolism was enhanced in TILs, which had also been observed in melanoma patient-derived tumour xenografts that grew into primary human melanoma tumours metastases in NSG mice. After comparing the types of FAs in the patient-derived tumour xenograft tumour interstitial fluid with those in solid organs or serum, researchers found that the levels of many types of FAs were significantly increased in the tumours (Zhang et al., 2017). Related research was based on an analysis of CD90.2+, which is homologous to CD45, in mice vaccinated and treated with FF (CD45.1 mice) or diluent (CD45.2 mice) performed to assess how increased FA catabolism affects CD8^+^ TILs. After splenocytes from the FF- or diluent-treated mice were transferred to other tumour-bearing mice, the former splenocytes significantly delayed tumour progression, which confirmed that enhanced FA catabolism increased the function of CD8^+^ TILs (Zhang et al., 2017). Other studies have reported that CD8^+^ T cells can enhance PPARα signal transduction, mitochondrial activation, and fatty acid catabolism to overcome hypoglycaemia and hypoxia in the TME. Fenofibrate can activate PPARα signal transduction and fatty acid catabolism, and when combined with PD-1 blockers, it showed a significantly increased therapeutic effect (Zhang et al., 2017; Desharnais et al., 2022).

Studies with preclinical mouse models of pancreatic ductal adenocarcinoma (PDAC) and human tumour specimens have shown that the progression of PDAC is characterized by TME enrichment with specific accumulating lipids. In the lipid-enriched TME, intrapancreatic CD8^+^ T cells are depleted, and therefore, their overall ability to metabolize available lipid substrates was decreased. In addition, the increase in lipid levels led to decreased mitochondrial function and induced FAO-mediated toxicity in CD8^+^ T cells in the pancreas and increased the cell death rate. Relevant experimental data showed that metabolic reprogramming of tumour-specific CD8^+^ T cells may be a strategy to promote their survival in a metabolically unfavourable TME, and they can be thus used to improve the clinical efficacy of immunotherapy (Manzo et al., 2020). Relevant lipidomic analysis have confirmed that the lipid composition of tumour-infiltrating CD8^+^ T cells changes significantly, with long-chain fatty acids mainly accumulating in the late stages of cancer. By determining the ability of CD8^+^ T cells to form LDs *in vivo* during tumour progression, researchers discovered that although more than 20% of CD8^+^ T cells infiltrating early lesions carried LDs, the percentage of these cells carrying LDs decreased significantly by the later experimental time points. Experimental data showed that infiltrating CD8^+^ T cells in advanced pancreatic intraepithelial neoplasia showed a limited capacity to store specific long-chain fatty acids in LDs, which may have accounted for increased lipotoxicity (Manzo et al., 2020). In obese mice of spontaneous breast tumour remission, ablation of T-cell STAT3 protein or treatment with FAO inhibitors reduced FAO, increased glycolysis and upregulated the function of TEFFs, which inhibited the development of the breast tumours, while PD-1 ligation to CD8^+^ T cells activated Stat3 to increase FAO, inhibit glycolysis and downregulate the function of TEFFs. In addition, researchers found that leptin enriched in breast adipocytes/adipose tissue downregulated the function of CD8^+^ T-cell effector factors by activating STAT3 in the FAO pathway and inhibited glycolysis (Zhang et al., 2020a).

Regulatory T cells (Tregs) secrete cytokines into the TME that inhibit the antitumour immune response and facilitate tumour cells escape immune surveillance. Tregs are CD4^+^ T cells that express FoxP3, a primary regulator of Treg development and function. It increases fatty acid uptake, oxidative phosphorylation, and FAO. Moreover, FoxP3 enhances the resistance of Tregs to lipotoxicity, such as that induced by the TME (Howie et al., 2017; Corn et al., 2020). Studies have shown that before many Tregs undergo apoptosis, they undergo severe oxidative stress, promoting immunosuppression and causing systematic Treg migration into the TME. In addition, Tregs proliferate and die rapidly; therefore, proliferating and apoptotic Tregs are simultaneously located in the TME (2). The lipid biosynthesis of Tregs in the TME is enhanced and is dependent on FAO. Therefore, it has been suggested that reducing the entry of Tregs into the TME by inhibiting their lipid metabolism may weaken their immunosuppressive effect. Studies have shown that the short-chain fatty acid propionic acid promotes the development of Treg cells, which may affect the development of tumours. Tregs can infiltrate a tumour, which leads to poor prognosis. Relevant studies have shown that Tregs promote the immune escape of tumour cells by inhibiting the induction of cytotoxicity by immune cells. In addition, Tregs cooperate with M2 macrophages and other tumour-promoting cells. For example, Tregs inhibit CD8^+^ T-cell secretion of IFN-γ, which can block the activation of fatty acid synthesis in M2 TAMs. In addition, through their effects on CD8^+^ T cells, Tregs promote the mitochondrial integrity of M2 TAMs. Therefore, the stability of Treg cell function in the TME is essential for TAMs to maintain an intracellular free fatty acid storage pool. Attenuating the *de novo* fatty acid synthesis pathway by targeting SREBP1 on which it depends may become an effective immunotherapy. In addition, studies have shown that inhibiting SREBP1 promotes the regeneration of CD8^+^ T cells, inhibits M2 TAMs, promotes the antitumour immune response, and enhances the efficacy of immune checkpoint inhibitors (ICIs) (Liu et al., 2019).

### Macrophages

Macrophages in the TME, also known as TAMs, are categorized mainly into two types. M1 macrophages mainly promote inflammation, which can contribute to inflammatory response maintenance and pathogen killing, while M2 macrophages play mainly an anti-inflammatory and a tumour-promoting role. M2 macrophages suppress inflammation and tissue repair, and their metabolism shifts to FAO and oxidative phosphorylation (Corn et al., 2020; Rosa Neto et al., 2021; Shi et al., 2022). Macrophages tend to switch between the M1 and M2 type throughout the TME (2). During tumorigenesis, macrophages tend to polarize into the M1 type. These M1 macrophages promote antitumour immune and inflammatory responses. As the tumour progresses into later stages, macrophages undergo reprogramming and switch into the M2 type, expressing anti-inflammatory factors to induce immunosuppression and promote tumour growth. Lipid metabolism plays an essential role in the polarization of M1 and M2 macrophages. The membrane remodelling of M1 macrophages and the synthesis of inflammatory mediators require the synthesis of many lipids. In M1 macrophages, SREBPs and FASN are important enzymes that regulate lipid biosynthesis. The former are crucial proteins, and the latter is a key enzyme. FASN plays an essential role in M1 induction. FAO exerts a significant influence on the activation of M2 macrophages. Relevant studies have shown that interfering with the lipid metabolism of macrophages can enhance the antitumour immune response by inhibiting CD36-mediated lipid uptake and FAO. Lipids are the key metabolites in the polarization of macrophages. M1 macrophages synthesize FAs as precursors for the synthesis of inflammatory mediators, while M2 macrophages do not relay on rapid lipid production because they participate in processes that reduce inflammation; therefore, they rely mainly on FAO. Generally, most macrophages in adipose tissue acquire the M2 phenotype. In the future, inflammation control in the adipose tissue of individuals with obesity may be realized by reprogramming macrophages into the anti-inflammatory type. Relevant studies have reported that TAMs polarize into the inflammatory M1 or tumorigenic M2 type when exposed to certain environmental stimuli. It is assumed that tumour-derived signalling induces lipid accumulation in macrophages and then induces macrophages to reach a tumour-promoting state characterized by the M2 phenotype. Macrophages were cultured *in vitro* with TES from mice with MFC gastric cancer. Treatment with 30% TES increased the lipid level in the macrophages by more than threefold. Further analysis showed that the expression of M2 markers (CD206, CD163, TGFβ, and Arg-1) was increased in macrophages treated with TES, while that of M1 markers (IL-12 and CD80) was decreased. Then, the researchers used the PI3K-γ inhibitor IPI-549 to examine the function of lipid-induced macrophages and found that the ablation of PI3K-γ inhibited the premigration or preinvasion of macrophages treated with TES or lipid mixtures, suggesting that selective abrogation of macrophage PI3K-γ may prevent the functional polarization of macrophages caused by tumour lipid accumulation. Based on the experimental results, the use of a PI3K-γ inhibitor normalized the lipid abundance in TAMs and reduced the tumor-promoting function of TAMs. Ultimately, it promoted T-cell-mediated cytotoxicity and inhibited MFC tumour growth (Luo et al., 2020).

Long-chain fatty acid (LCFA) oxidation plays an important role in IL-4-mediated macrophage polarization [M (IL-4)]. The polarization of macrophages is related to the enhancement of fatty acid oxidation (O'Neill et al., 2016; Van den Bossche et al., 2017). Carnitine palmitoyl transferase-1 (CPT-1) facilitates the binding of long-chain fatty acyl CoAs to carnitine, promoting the carnitine uptake by the mitochondrial matrix, where it undergoes oxidation. A study by Divakaruni, A. S et al. confirmed that high concentrations of etomoxir (carnitine palmitoyltransferase-1 inhibitor) inhibited macrophage (IL-4) polarization independent of CPT-1 activity. High concentrations of etomoxir inhibited adenine nucleoside transferase and disrupted CoA homeostasis. Cytoplasmic CoA is essential for processes such as lipid synthesis, vesicular trafficking, fatty acid oxidation, and desaturation (Pietrocola et al., 2015). Long-chain fatty acyl CoAs are highly specific and play unique regulatory roles in lipid synthesis and degradation (Neess et al., 2015). Research has shown that the availability of CoA may be a limiting and targetable factor in regulating M (IL-4) polarization (Divakaruni et al., 2018).

TAMs play vital roles in the occurrence and development of tumours. Researchers used an adipocyte fatty acid binding protein (A-FABP) deficient (A-FABP^−/−^) mouse model to evaluate the effect of A-FABP deficiency on the growth and metastasis of breast tumours. E0771 tumour cells from C57BL/6 mouse models of breast cancer were injected *in situ* into the mammary fat pads of A-FABP^−/−^ mice and their wild-type (WT) littermate mice for observation and research, and the expression of A-FABP in TAMs promoted the development of breast cancer. Then, different types of tumour cells, including mouse breast tumour cells (MMT cells) and colon cancer cells (MC38 cells), were used to further evaluate the tumour growth and metastasis in WT and A-FABP−/− mice. Regardless of the expression of A-FABP in the tumour cells, A-FABP deficiency in the host cells inhibited tumour growth and metastasis. The tumorigenic effect of A-FABP is mediated by TAMs and the expression of A-FABP in TAMs regulates the NFκB/miR-29b signal transduction pathway and promotes IL-6/STAT3 signal transduction in tumour cells; therefore, macrophages in advanced tumours show preferential expression of A-FABP and support tumour progression through IL-6/STAT3 signal transduction (Hao et al., 2018; Vitale et al., 2019). Studies have shown that cholesterol metabolism is closely related to changes in macrophage polarization (Vitale et al., 2019). ABCA transporter subfamily proteins, such as ABCA1 and ABCA9, are essential for maintaining cellular cholesterol homeostasis (Piehler et al., 2002; Plummer et al., 2021). Genetic deletion of the ABC transporter can block the outflow of membrane cholesterol from macrophages and inhibit tumour progression (Goossens et al., 2019). Researchers have established a mouse CRC-PC (colorectal cancer and peritoneal carcinomatosis) model by transplanting the MC38 and CT26 colorectal cancer cell lines. The results showed that 5Aza significantly inhibited the growth of the MC38 tumours in a dose-dependent manner, and the survival rate of the mice with peritoneal metastasis was significantly prolonged by the 5Aza treatment, results that were confirmed with the CT26 cell model mice. Then, by examining the changes in the number of macrophages in visceral fat, the researchers found that the infiltration of all types of macrophages in the visceral fat of the 5Aza-treated mice had decreased, the proportion of M1 macrophages had increased significantly over time, and the proportion of M2 macrophages had decreased significantly. Further research revealed that 5Aza promoted macrophage polarization and subsequent T-cell activation. Then, through a Drug Affinities Target Stability (DARTS) test, the researchers found that 5Aza protected ABC A9 and concluded that 5Aza targeting ABC A9 promoted the accumulation of cholesterol in macrophages, thereby increasing the expression of p65-dependent IL-6 and activating T cells to inhibit CRC-PC development (Shi et al., 2022).

ATP binding cassette transporter G1 (ABCG1) promotes cholesterol outflow from cells and regulates intracellular cholesterol homeostasis. To determine whether ABCG1 affects tumour growth, researchers subcutaneously injected MB49 bladder cancer or B16F1 melanoma cells into 7- to 10-week-old Abcg1^−/−^ or control C57BL/6 (WT) mice. The study found that Abcg1^−/−^ mice bearing MB49 tumours survived longer than WT mice bearing MB49 tumours. Generally, ABCG1 deficiency *in vivo* inhibited tumour growth and increased animal survival. Then, researchers adopted a bone marrow chimaera and found that ABCG1 deficiency changed the balance between tumour-promoting immune cells and antitumour immune cells in the TME. Further research revealed that the loss of ABCG1 inhibited tumour growth by regulating the survival and phenotype acquisition of macrophages in tumours. For example, ABCG1led to the transformation of the remaining macrophages into the antitumour M1 phenotype. Therefore, ABCG1 may be a therapeutic target for cancer (Sag et al., 2015; Hoppstädter et al., 2021).

### DC cells

DC cells are antigen-presenting cells. Mutated cells in the body release tumour antigens after death. After antigen-presenting cells such as DCs are activated, they present tumour antigens to CD4^+^ T cells or CD8^+^ T cells, which are activated and proliferate into tumour-specific CTLs, enhancing the cytotoxic effects of the CD8^+^ T cells. DCs need to be activated before they can activate T cells, and AcCoA is the key DC-activating enzyme (Coleman et al., 2020). DCs are regulated by lipid metabolism. For example, bone marrow-derived DCs stimulated by lipopolysaccharide (LPS) amplify FA biosynthesis and then promote the expansion of the Golgi apparatus and endoplasmic reticulum to promote the production of cytokines. Herber DL et al. found that the levels of triglycerides in DCs of tumour-bearing (TB) mice and cancer patients were significantly increased. Upregulation of scavenger receptor (SR) A increases extracellular lipid uptake and leads to lipid accumulation. The upregulation of Msr1 (CD204) profoundly contributes to lipid accumulation in DCs in the cancer context. The ability of DCs with high lipid content to present antigens is significantly reduced. Researchers treated DCs with the acetyl-CoA carboxylase (ACC) inhibitor 5-(tetradecycloxy)-2-furoic acid (TOFA). After treatment, the number of DC lipids decreased to normal levels, and DC functional activity was restored. Further research has shown that the combination of this inhibitor and cancer vaccines significantly enhanced vaccine effectiveness (Herber et al., 2010).

Relevant studies have shown that the high lipid content in DCs blocks the expression of major histocompatibility complex (MHC) I peptide complexes, thus reducing the cross-presentation of antitumour antigens. The accumulation of toxic lipid peroxidation byproducts in the TME leads to endoplasmic reticulum stress in tumour-infiltrating dendritic cells by activating the IRE1 α-XBP1 axis, which can promote the synthesis of lipid species, especially TAG, and increase the LD content. In addition, a high lipid level hinders the presentation of tumour antigens and the activation of antitumour T cells (Cubillos-Ruiz et al., 2015; Ma et al., 2020). Researchers incubated DP7-C/antigen with immature DCs, pretreated bone marrow-derived cells (BMDCs) with DP7-C and then added antigen peptide to the BMDCs or added antigen peptide and DP7-C to the BMDCs. They evaluated whether DP7-C and the antigen peptide entered BMDCs in the form of complexes and whether this combination treatment led to significant changes in the cell survival rate. The results showed that cholesterol-modified antimicrobial peptide DP7 (DP7-C) with low toxicity induced DC maturation and cytokine secretion, increasing the efficiency of antigen presentation and increasing the effect of a DC vaccine. Compared with a mouse model treated with DC7-C/antigen alone, a mouse model treated with DC7-C/antigen and a mouse model subjected to antigen pulsing showed more robust antitumour responses (Zhang et al., 2020b). A study of B16-F10 melanoma tumour-bearing model mice found that PD-1 ablation in myeloid cells induced effective antitumour immunity by reducing the accumulation of MDSCs and promoting the differentiation of proinflammatory and effector monocytes/macrophages and DCs, resulting in an enhanced effector T-cell response. Cholesterol promotes the expansion of myeloid cells and macrophages and DC differentiation (Westerterp et al., 2017) and promotes cell antigen-presenting function (Anderson and Roche, 2015), which may be the critical mechanism by which PD-1 blocks cell cycle progression and induces antitumour effects (Strauss et al., 2020).

### NK cells

NK cells are innate lymphoid cells widely distributed in the blood, peripheral lymphoid tissue, liver, and spleen. Through its IgGFc receptor on its surface, an NK cell can kill tumour target cells through ACC (Chen and Sui, 2021). As an essential regulator of tumour immune surveillance, NK cells recognize lipid antigens presented by the nonclassical MHC-class I molecule CD1d (Tiwary et al., 2019; Devillier et al., 2021), and its adsorption capacity is affected by vascular endothelial growth factor (VEGF). The cellular metabolism of NK cells is dynamically regulated and strongly affects their response. Metabolic disorders are associated with defects in the NK cell response in diseases such as obesity and cancer. Related studies have revealed that SREBP plays a new role in *de novo* polyamine synthesis by promoting the expression of cMyc in NK cells. This finding provides a relevant basis for the previously cited study showing that a cholesterol-rich TME inhibits SREBP activation and drives lymphocyte dysfunction (O'Brien et al., 2021). In relevant experiments, C57BL/6J and NKdele mice were injected with diethylnitrosamine to induce liver tumour formation, and then, the mice were fed a standard diet (ND) or high cholesterol diet (HCD) to induce the production of serum cholesterol to high levels. Hep1-6 (mouse liver cancer) cells were implanted in the C57BL/6J and NKdele mice on an ND or HCD, and the growth of the xenograft tumours and lung metastasis development was monitored. Then, NK cells were isolated from mice. By analysing the changes in the cholesterol content, lipid raft formation, immune signalling pathway activation, and overall function of the NKs were evaluated. The results showed that compared with the ND-fed mice, the C57BL/6J mice fed a HCD developed fewer and smaller liver tumours and lung metastasis after injection with diethylnitrosamine after implantation of the Hep1-6 cells, and the number of NK cells in the mice fed a HCD increased. Further analysis revealed that cholesterol accumulated in the NK cells and promoted their effects on liver cancer cells. The paper concluded with the suggestion that hepatocellular carcinoma (HCC) can be treated by increasing the cholesterol uptake by NK cells (Qin et al., 2020). Innate lymphocytes that expressed both the NK cell surface marker CD56 and the T-cell surface marker TCRαβ-CD3 complex are called natural killer T cells (NKTs). Type I and Type II NKTs regulate the immune response during tumour progression. Type I NKTs enhance the antitumour immune response, while Type II NKTs exert the opposite effects (Liu et al., 2021b). Lipids are essential for the development of NKTs. Relevant studies have shown that changes in lipids can directly or indirectly regulate NKT-dependent immune function. Activation of NKTs requires an increase in lipid biosynthesis (Fu et al., 2020). However, excessive lipids interfere with CD1d-lipid-antigen complex binding to TCRs, hindering the activation of NKTs. Because lipid changes affect NKTs and tumour immunity, lipid reactive NKTs have become crucial immunomodulators of tumour immunity in clinical mouse models (Tiwary et al., 2019).

### PMNs

PMNs derived from granulocyte/macrophage precursors in the bone marrow are essential effector cells involved in inflammation or the allergic inflammatory response. Researchers identify LDs by staining cells with Nile red dye. In PDAC, the intratumoral infiltration of PMNs has been found to induce histological changes in tumour cells and regulate tumour growth patterns. α-SMA downregulation and LD remodelling in PMN-dense regions is a manifestation of metabolic reprogramming (Mayer et al., 2018). Apolipoprotein A-1 (apoA-I) is the main protein form of high-density lipoprotein, and it inhibits the formation of blood vessels in tumours (Sirni? et al., 2017). In addition, apoA-I inhibits neutrophil activation, migration, and adhesion. Moreover, relevant studies have shown that combining apoA-I and neutrophils may be a practical prognostic index for HCC patients undergoing transcatheter arterial chemoembolization. By studying the prognostic value and potential immune mechanism of the neutrophil apolipoprotein A ratio (NAR) in HCC patients receiving transcatheter arterial chemoembolization in two independent cohorts, researchers found that NAR showed prognostic performance similar to that obtained with the Cancer of the Liver Italian Program score, and when the score was adjusted by considering the NAR, the prediction for overall survival and survival rate at a specific time point was more accurate (Chen et al., 2021). The generation of PMNs requires long-range intercellular communication continuously mediated by soluble or membrane-binding factors on primary tumours (Liu and Cao, 2016; Peinado et al., 2017), and intercellular communication synapsis formation and timing are controlled by cholesterol. Therefore, cholesterol can regulate the efficiency of intercellular communication throughout all the stages of neutrophil generation in the TME and other environments. Researchers have speculated that the cellular cholesterol burden at premetastatic sites may tightly regulate the efficiency of cancer cell extracellular vesicle (EV)-mediated intercellular communication with target cells at these sites. Further experiments revealed that the communication between prostate cancer (PCa) EVs and bone marrow resident cells depended on cholesterol. The experimental results showed that bone marrow, a vital PMN-generating target tissue, resisted prostate cancer EV signal transduction when the cholesterol level in myelocytes was reduced (Henrich et al., 2020). DCs can cross-present tumour-related antigens, but the cross-presentation performance of DCs in tumours is impaired, and these DCs may be targets for tumour immunotherapy.

### MDSCs

MDSCs are precursors of DCs, macrophages, and granulocytes. They inhibit the immune response and play essential roles in tumorigenesis. MDSCs constitute a group of highly heterogeneous immature myeloid cells that are usually classified into two main subgroups: polymorphonuclear myeloid-derived suppressor cells and monocyte (M) MDSCs (Gabrilovich, 2017), which inhibit adaptive immunity and hinder the effect of anticancer therapy (Porta et al., 2020). MDSCs undergo metabolic reprogramming, switching from glycolysis to FAO during tumorigenesis, and they exhibit oxidative phosphorylation when tumour lipid accumulation. As the uptake of exogenous FA by MDSCs in tumours increases, their immunosuppressive activity on T cells also increases. For example, MDSCs with lipid overload showed a more potent immunosuppressive effect on CD8^+^ T cells. Some key enzymes in MDSCs consume essential amino acids required for T cell function and proliferation. In tumours, the inhibitory ability of MDSCs is enhanced by the upregulation of lipid metabolism genes induced by unsaturated FAs, including diacylglycerol acyltransferase. In addition, the signalling pathways in lipid metabolism in tumours affect the role of MDSCs; these pathways include the LXR, PPARs, AMPK, PI3K/AKT/mTOR, STAT, TIPE family, and prostaglandin E2 (PGE2) pathways. In Lewis lung cancer, bone marrow-derived TAMs upregulated the production of COX2 and prostaglandins, which was positively correlated with an increase in tumour angiogenesis and MDSC expansion (Poczobutt et al., 2016; Porta et al., 2020). Related studies reported that tumour-derived PGE2 induced nuclear accumulation of P50 NF-κB in M-MDSCs, shifted their response from IFNγ-to NO-mediated immunosuppression, and reduced TNFα expression. In addition, the same study also revealed that inhibiting PGE2/P50/NO axis activation suppressed the inhibitory functions of MDSCs and restore the effects of anticancer immunotherapy (Porta et al., 2020). Apolipoprotein E (ApoE) is a secreted protein involved in lipoprotein metabolism. LXRβ and LXRα are members of the nuclear hormone receptor transcription factor family, and these proteins drive the transcriptional activation of ApoE, LXR, and their gene targets. ApoE plays an essential role in regulating MDSC abundance and antitumour immunity. Researchers have studied the role of ApoE in LXR-mediated MDSC deletion by analysing the effect of LXR treatment on tumour MDSC abundance and ApoE consumption in tumour cells after ApoE gene inactivation in stroma and immune system. LXR treatment failed to significantly reduce the number of tumour MDSCs or tumour volume in ApoE^−/−^ mice without ApoE B16F10 tumour cells compared with WT mice. The LXR transcription target gene ApoE mediates the depletion of LXR-dependent MDSCs. Additional experiments confirmed that the amplified MDSCs showed immunosuppressive activity despite ApoE depletion (Tavazoie et al., 2018).

Relevant studies have shown that by producing truncated lipids that are oxidized, PMN-MDSCs block the cross-presentation of DCs without affecting direct DC presentation of antigens; this process depends on myeloperoxidase (MPO). Experiments confirmed that MPO-deficient PMN-MDSCs did not affect cross-presentation by DCs, and the cross-presentation of tumour-related antigens by DCs *in vivo* was increased in MDSC-null or tumour-bearing MPO-KO mice. Experiments showed that MPO-driven lipid peroxidation, a possible noncellular autonomous mechanism in PMN-MDSCs, inhibited the cross-presentation of antigens by DCs. Therefore, researchers proposed that MPO, a potential therapeutic target, can be used to increase the efficacy of immunotherapy (Ugolini et al., 2020). Polymorphonuclear myeloid-derived suppressor cells are pathologically activated neutrophils that are essential for regulating the immune response to cancer. Lipid metabolism is a crucial regulator of PMN-MDSCs (Veglia et al., 2019; Chen et al., 2021). A study found that fatty acid transporter 2 (FATP2) controlled the inhibitory activity of PMN-MDSCs. In addition, the study found that mouse and human PMN-MDSCs upregulated FATP2. In an analysis with WT and FATP2-knockout LLC tumour-bearing mice, LC‒MS showed that compared with the PMN-MDSCs of the WT mice, the PMN-MDSCs isolated from the spleens of the FATP2-knockout LLC tumour-bearing mice showed reduced amounts of total triglycerides, especially triglycerides carrying arachidonic acid. Hence, the immunosuppressive activity of PMN-MDSCs mediated by FATP2 can be considered a target for selectively inhibiting the function of PMN-MDSCs and increasing the efficiency of cancer treatment (Veglia et al., 2019).

PMN-MDSCs play an important role in the regulation of immune responses in cancer. Condamine, T et al. proposed that oxidized low-density lipoprotein (LDL) receptor 1 (OLR1) can be a specific marker of immune responses by PMN-MDSCs. They observed significant upregulation of OLR1 in many types of cancer. The upregulation of OLR1 expression was positively correlated with clinical stage in certain cancers, such as bladder cancer and clear cell kidney cancer. In patients with prostate adenocarcinoma, colorectal adenocarcinoma or bladder cancer, upregulated OLR1 expression was positively correlated with tumour size. Hence, lectin-type oxidized LDL receptor 1 (LOX-1) encoded by OLR1 on PMN-MDSCs can be a target for treatment (Condamine et al., 2016).

### Obesity and antitumour immunity

Obesity is a disease characterized by excessive adipose tissue (Rathmell, 2021). Studies have indicated that obesity is related to many cancers, such as pancreatic cancer, endometrial cancer, liver cancer, and renal cell cancer (Wangpaichitr et al., 2012; Zhang et al., 2020a; Moukarzel et al., 2022). In addition, the recurrence of certain cancers unrelated to obesity can also be affected by obesity, such as breast cancer. WAT stores triglycerides and functions as an endocrine organ (Coleman et al., 2020). Adipokines in the obesity context include adiponectin, leptin, and proinflammatory cytokines. The abnormal signal transduction of these cytokines is related to the impairment of immune function. In addition, the activity of immune cells is regulated by obesity-induced inflammation. For example, leptin promotes the activation of T cells and the formation of MDSCs. Blocking leptin receptors can increase the ICI response. Studies have shown that immunosuppression caused by chronic inflammation in the obesity context may promote tumour growth, and it can also increase the sensitivity of specific cell populations to ICI treatment. It is generally believed that moderate caloric restriction plays a significant role in fighting cancer. Some studies have shown that caloric restriction partially reversed the immune dysfunction caused by obesity and enhanced immune surveillance. During intermittent fasting, the leptin level of the body circulates in a different way, promoting the anticancer effect of Cr and providing enough leptin to support immune cells. Studies have found that intermittent fasting reprogrammed TAMs and enhanced the cytotoxicity of CD8^+^ T cells to promote an antitumour immune response. Therefore, the combination of intermittent fasting and immunotherapy may lead to greater treatment efficacy (Coleman et al., 2020). Wang et al. proposed that obesity should be regarded as a potential mediator of immune dysfunction and tumour progression. They proved that obesity led to immune system ageing, tumour progression and PD-1-mediated T-cell dysfunction in a variety of species and tumour models (B16 melanoma, 4T1 breast cancer, etc.). Leptin played a clear role in this process. The efficacy of PD-1/PD-L1 inhibitors in diet-induced obese mice (DIO, 60% fat diet) was significant, and this effect has been verified in clinical cancer patients (Wang et al., 2019).

Obesity affects the host environment, promoting inflammation. Generally, adipose tissue comprises adipocytes and cells related to anti-inflammatory and immune responses. These cells include helper T cells, regulatory T cells, M2 macrophages, and eosinophils. They work together to maintain a metabolic balance and prevent metabolic dysfunction. Long-term intake of calories that exceed bodily needs coupled with functional remodelling of adipose tissue leads to the establishment of a proinflammatory environment. The types of adipokines produced change from anti-inflammatory adiponectin production to proinflammatory leptin, and the concentration of local proinflammatory mediators also increases. Leukocytes aggravate metabolic disorders and the systemic inflammatory response when inflammation persists (Desharnais et al., 2022; Moukarzel et al., 2022).

Given that chronic inflammation is associated with cancer risk (Grivennikov et al., 2010), Quail et al. fed mice on different diets and analysed the effect on cells by performing flow cytometry and immunoassays. Ultimately, they found that obesity relied on GM-CSF and IL5, which increased the number of in lung neutrophils. This outcome was related to enhanced cancer metastasis. Losing weight effectively reversed this outcome (Quail et al., 2017).

The metabolism of CD8^+^ T cells in various states is very sensitive to external factors. Obesity is an external factor that exerts a significant impact on CD8^+^ T cells. Among these factors, the proinflammatory adipokine leptin promotes T-cell immune functions, allowing T cells to effectively participate in the host immune response (Zhang et al., 2020a).

## Discussion

To increase the effectiveness of immunotherapy, many studies have combined lipid metabolism and immunotherapy. Some teams have been devoted to studying the role of lipid metabolism in the clinical prognosis prediction of immunotherapy. Studies have revealed that the hypermutation status of lipid metabolism pathway components was associated with significantly prolonged progression-free survival in NSCLC, suggesting that these factors can be measured to predict NSCLC patients receiving ICIs (Cheng et al., 2021). In addition, related studies have reported a lipid metabolism-related gene prognosis prediction model and that stratification of lung adenocarcinoma patients into high-risk groups and low-risk groups helped predict the effectiveness of immunotherapy. Considering that previous studies have constructed clinical prognosis models of oral squamous cell carcinoma, glioblastoma multiforme, and CRC based on lipid metabolism, the impact of lipid metabolism on the prognosis of various types of cancer immunotherapy will be further studied in the future (Zhang et al., 2022). We have listed some proven cases of an effective combination of lipid metabolism factors and immunotherapy in Table 2. Research groups have also found that obesity affects the efficacy of anti-PD-1 therapy in cancer patients and have proposed that obesity-related factors are biomarkers for certain cancer immunotherapies. They experimentally found that obesity accelerated T-cell senescence, resulting in increased PD-1 expression and dysfunction in multiple species and tumour model (Wangpaichitr et al., 2012). Therefore, further study into the impact of obesity and dyslipidaemia on cancer immunotherapy is of great importance (Bleve et al., 2020). Analysing many articles in the literature (Kawalekar et al., 2016; Angelin et al., 2017; Pacella et al., 2018; Wang et al., 2018; Lin et al., 2020; Yu et al., 2021), we found that lipids play contradictory roles in the TME, as they can both support antitumour immune responses and promote tumour immune responses, suggesting that simply inhibiting or stimulating one lipid metabolism pathway in the TME is not a sufficient therapeutic strategy. However, most current therapies targeting lipid metabolism in tumour-infiltrating immune cells focus on a single type of cell or metabolic pathway, and future studies that take into account the paradoxical role played by lipids may optimize existing cancer treatments (Yu et al., 2021).

**TABLE 2:** A proven combination of lipid metabolism and immunotherapy.

Target protein	Inhibition	Combined therapy	Tumor model	References
SCAP/SREBPs	Tamoxifen	Anti-PD-1	MC38, B16	Lim et al. (2021)
CD36	Genetic knockdown	Anti-PD-1	B16	Ma et al. (2021)
ACAT	Avasimibe	Anti-PD-1	B16	Yang et al. (2016), Harel et al. (2019)
FATP2	Lipofermata	Anti-CTLA4	LLC, TC-1	Veglia et al. (2019)
		Anti-CSF1R		
PCSK9	Evolocumab, Alirocumab	Anti-PD-1	B16, MC38, 4T1, CT26	Liu et al. (2020)
SQLE	Genetic knockdown	ICB	GBM	Ye et al. (2023)
LDH	FX-11	Anti-PD-1	4T1	Gong et al. (2021)
ACC	TOFA	Cancer vaccines	EL-4	Herber et al. (2010)

SREBPs, sterol-regulatory-element-binding proteins; SCAP, SREBP-cleavage-activating protein; MC38, colon adenocarcinoma; B16 melanoma; CD36 encoding a scavenger receptor responsible for long-chain fatty acid and oxidized low-density lipoprotein uptake; ACAT, Recombinant Acetyl Coenzyme A Acetyltransferase; FATP2 fatty acid transport protein 2; LLC, lewis lung carcinoma; TC-1, HPV16 E6/E7-expressing tumor cell line; PCSK9, Proprotein Convertase Subtilisin/Kexin Type 9; 4T1 breast carcinoma; CT26, colon carcinoma; SQLE, squalene monooxygenase; ICB, Immune-checkpoint blockade; GBM, Glioblastoma; LDH, lactate dehydrogenase; ACC acetyl-CoA carboxylase; EL-4 lymphoma.

## Figures and Tables

**Table:** 

**NSCLC**	non-small cell lung cancer
**DCs**	dendritic cells
**TAMs**	tumor-associated Macrophages
**PMNs**	Polymorphonuclear neutrophils
**MDSCs**	Myeloid-derived suppressor cells
**FAs**	fatty acids
**SREBPs**	sterol-regulatory element binding proteins
**LXR**	liver X receptor
**PPARs**	peroxisome proliferators-activated receptors
**AcCoA**	acetyl coenzyme A
**FAO**	fatty acid oxidation
**CPT1**	Carnitine palmitoyltransferase 1
**FASN**	fatty acid synthase
**WAT**	white adipose tissue
**LDs:**	lipid droplets
**TILs**	tumor infiltrating lymphocytes
**CTLs**	cytotoxic T cells
**TEFFs**	CD8^+^ effector T cells
**ACAT**	cholesterol acyltransferase
**PDAC**	Pancreatic ductal adenocarcinoma
**Tregs**	Regulatory T cells
**ICIs**	Immune checkpoint inhibitors
**LCFA**	Long-chain fatty acid
**CPT-1**	Carnitine palmitoyl transferase-1
**A-FABP**	adipocyte fatty acid binding protein
**WT**	wild-type
**ABCG1**	ATP binding cassette transporter G1
**MHC**	major histocompatibility complex
**BMDC**	bone marrow-derived cells
**ND**	normal diet
**HCD**	high cholesterol diet
**HCC**	hepatocellular carcinoma
**NKTs**	natural killer T cells
**apoA-I**	Apolipoprotein A-1
**NAR**	neutrophil apolipoprotein A ratio
**EV**	Extracellular vesicles
**PGE2**	prostaglandin E2
**ApoE**	Apolipoprotein E
**MPO**	myeloperoxidase
**FATP2**	fatty acid transporter 2
