# Corrigendum: Lipid metabolism and tumor immunotherapy

**DOI:** 10.3389/fcell.2024.1349379

**Published:** 2024-01-26

**Authors:** Yue Wang, Zongjin Guo, Adamu Danbala Isah, Shuangwei Chen, Yongfei Ren, Huazhong Cai

**Affiliations:** ^1^ School of Medicine, Jiangsu University, Zhenjiang, China; ^2^ Cancer Institute of Jiangsu University, Affiliated Hospital of Jiangsu University, Zhenjiang, China; ^3^ Department of Emergency, Affiliated Hospital of Jiangsu University, Zhenjiang, China; ^4^ Department of Interventional Radiology, The University of Hong Kong-Shenzhen Hospital, Shenzhen, China

**Keywords:** lipid metabolism, tumor microenvironment, immunotherapy, combination therapy, immune cells

In the published article, there was an error regarding the **Affiliation(s)** for [Yue Wang]. As well as having affiliation(s) [1, 2], they should also have “*Department of Emergency, Affiliated Hospital of Jiangsu University, Zhenjiang, China*.”

The authors apologize for this error and state that this does not change the scientific conclusions of the article in any way. The original article has been updated.

